# Protocol of a cluster randomized trial to investigate the impact of a type 2 diabetes risk prediction model on change in physical activity in primary care

**DOI:** 10.1186/s12902-018-0299-2

**Published:** 2018-10-16

**Authors:** Esther Jacobs, Miguel Tamayo, Joachim Rosenbauer, Matthias B. Schulze, Oliver Kuss, Wolfgang Rathmann

**Affiliations:** 1 Institute for Biometrics and Epidemiology, German Diabetes Center (DDZ), Leibniz Center for Diabetes Research at Heinrich Heine University Düsseldorf, Auf’m Hennekamp 65, 40225 Düsseldorf, Germany; 2 German Center for Diabetes Research (DZD), 85764 München-Neuherberg, Germany; 3 The Association of Statutory Health Insurance Physicians North Rhine, Tersteegenstraße 9, 40474 Düsseldorf, Germany; 4 German Institute of Human Nutrition Potsdam-Rehbruecke (DIfE), Arthur-Scheunert-Allee 114-116, 14558 Nuthetal, Germany; 5 Institute of Medical Statistics, Düsseldorf University Hospital and Medical Faculty, Heinrich Heine University Düsseldorf, Düsseldorf, Germany

**Keywords:** Risk score, Prevention, Type 2 diabetes, Physical activity, Behavior, Cluster randomized controlled trial

## Abstract

**Background:**

Little evidence exists on the impact of diabetes risk scores, e.g. on physicians and patient’s behavior, perceived risk of persons, shared-decision making and particularly on patient’s health. The aim of this study is to investigate the impact of a non-invasive type 2 diabetes risk prediction model in the primary health care setting as component of routine health checks on change in physical activity.

**Methods:**

Parallel group cluster randomized controlled trial including 30 primary care physicians (PCPs) and 300 participants in the region of Düsseldorf and surrounding urban and rural municipalities, West Germany. On cluster level, PCPs will be randomized into intervention or control group using a biased coin minimization technique. Participants in the control group are going to have a routine health check “Check-up 35” which is recommended biannually for all people ≥35 years of age in Germany. In the intervention group, the routine health check is expanded by usage of a non-invasive diabetes risk prediction model (German Diabetes Risk Score). Primary outcome is change in physical activity after 1 year. Secondary outcomes include aspects of targeted counseling, motivation of participant’s to change lifestyle, perceived and objectively measured diabetes risk, acceptance of diabetes risk scores, quality of life, depression and anxiety. Patients will be followed over 12 months. Hierarchical or mixed models will be conducted, including a random intercept to adjust for cluster, the respective baseline value, and covariates to compare the groups.

**Discussion:**

This pragmatic cluster randomized controlled trial will enhance our knowledge on the clinical impact of diabetes risk scores for the first time in the real-life primary health care setting.

**Trial registration:**

ClinicalTrials.gov NCT03234322, registered on July 28, 2017.

## Background

Studies on lifestyle interventions with participants of high risk for type 2 diabetes have shown that modifiable risk factors, such as physical inactivity and overweight can be reduced, thus, diabetes can be postponed or even prevented [1, 2]. For practical implementation of such programs, the major challenge is the identification of high-risk individuals. Primary care physicians (PCPs) are the first point of contact for health-related issues and have comprehensive knowledge about the medical condition of the person and his family, thus, they are the ideal contact point to identify persons at risk. Up to now, the screening process differs between PCPs, meaning the selection of people with increased diabetes risk is based on the individual decision to combine different screening steps. These include an initial screening based on single risk factors (e.g. age, weight, sex, Body-Mass-Index (BMI)) or diabetes risk scores including multiple risk factors as well as the measurement of blood glucose (fasting plasma glucose test, oral glucose tolerance test (OGTT), hemoglobin A1c test (HbA1c)) to determine the diabetes risk and glucose metabolic disorders [3].

Diabetes risk scores are predictive models to estimate the probability for an individual to develop diabetes within a defined time period. Those scores without laboratory measures (non-invasive risk scores) are more suitable for application at the population level, since they mostly include simple questions regarding anthropometric and lifestyle factors [4]. It has been proposed that using diabetes risk scores as first step of diabetes screening is more practical than blood glucose tests, as the latter are time consuming and costly [5]. Hence, the interest in the development of diabetes risk scores is still unabated. In the last years, several hundred diabetes risk prediction models were developed worldwide [6, 7]. However, little evidence exist on the impact of diabetes risk scores, i.e. on physicians and patient’s behavior, perceived risk of persons, shared-decision making in order to make an informed decision and particularly patient health [5, 7]. To date, only one study by Godino et al. [8] has been published, which assessed the effect of communicating an estimate of genetic or phenotypic risk of type 2 diabetes on physical activity. In this study, several modes of communication were used to inform the participants about their individual risk, including face-to-face counseling, telephone conversations, and printed materials [9]. However, uncertainty still exist on the impact of diabetes risk scores in the primary health care setting.

### Aim

Given the rapid development of diabetes risk scores and a simultaneous reluctance of PCPs to implement diabetes risk scores in everyday practice [10], there is an urgent need to expand our knowledge of the impact of diabetes risk scores in the primary health care setting. Thus, the aim of the study is to investigate the impact of a non-invasive risk prediction model in the primary health care setting as component of routine health checks. To prevent contamination effects between participants and also possible dilution effects of PCPs counseling strategy, the study is designed as cluster randomized trial, thus, PCPs will be objects of randomization.

### Objectives

Our primary objective is to investigate whether the application of the diabetes risk prediction model on individual level including an oral explanation of the result in the physician-patient consultation as component of the routine health checks in primary care will improve physical activity of individuals 1 year after the routine health check. Our secondary objectives are to determine, if the intervention will improve the counseling process regarding preventive strategies on balanced nutrition, body weight reduction and smoking secession, lead to improved shared decision making, and increase the motivation to change lifestyle. Furthermore, the aims are to evaluate whether the risk score leads to changes in Body Mass Index (BMI), quality of life, level of depression and anxiety, the perceived risk of developing diabetes as well as change on the individual diabetes risk. A final goal is how the application of a diabetes risk prediction model will be accepted as instrument for routine use in clinical practice.

## Methods/design

### Study design

The study is a pragmatic blinded parallel group superiority cluster randomized controlled trial. Clusters are PCPs (general practitioners, medical practitioners and internists working as general practitioners) located in the region of Düsseldorf and surrounding urban and rural municipalities in the federal state of North Rhine-Westphalia in Germany. They will be randomized to intervention or control group by minimization technique with a 1:1 allocation ratio [11].

### Recruitment of PCPs and participants

PCPs will be invited by the Association of Statutory Health Insurance Physicians North Rhine and the German Diabetes Center in Düsseldorf, both located in North Rhine-Westphalia, Germany. All PCPs with and without further training in diabetology according to German Diabetes Association standards that provide the routine health check can participate in the study, except PCPs who treat exclusively patients with private insurance or diabetes patients only. On invitation, all PCPs will receive an information letter including all aspects of the study with a consent form and baseline questions needed for randomization. Those interested to participate will send their consent and the answers to the baseline questions to the study center at the German Diabetes Center. All PCPs willing to participate will be consecutively randomized into one of the study groups until the target of 30 PCPs is achieved.

Thereafter, the PCPs will start to recruit ten people within a maximum of 2 years. To prevent selection bias, PCPs are constrained to recruit consecutively people who fulfil the following inclusion criteria: Appointment for the routine health check, statutory health insurance, age of > 35 years, and a BMI of ≥27 kg/m^2^. People are not eligible if they have been diagnosed with type 1 or type 2 diabetes or had already at least one measurement of abnormal blood glucose level (fasting glucose ≥126 mg/dl or 2 h oral glucose tolerance test (oGTT) ≥ 200 mg/dl or glycated hemoglobin (HbA1c) ≥6,5%) before the routine health check. Further exclusion criteria are no sufficient German language skills to fill out the questionnaires, presence of an incurable disease with a prognosis of less than 1 year, severe mental illness or dementia, and a severe underlying disease, which largely impairs physical activity. Further excluded will be pregnant women and people who participated in another clinical study 30 days before study inclusion.

### Intervention

The intervention will be integrated into a routine health check (Check-up 35), which all people in Germany with statutory health insurance > 35 years are entitled to every 2 years. The routine health check (Check-up 35) is conducted on two separate days and will be extended only with questionnaires in both groups, meaning that no study-specific additional measurements will be taken.

On the first day of the Check-up 35, the medical history is obtained and medical examination (complete physical examination and laboratory tests of blood and urine) is carried out. On the second day of the health check, which is usually a few days after the first day, a consultation takes place in which the results of the health check and preventive opportunities are discussed.

In the actual study, all people entering the PCP practice with an appointment of a routine health check and fulfilling the inclusion criteria will be informed about the study and receive an information letter including the consent form on the first day. They will enter the study when they give informed consent. On the second day of the routine health check, participants of both groups will receive the baseline questionnaire that can be filled in during waiting time. This questionnaire contains, among others, sociodemographic information, contact details, and questions on lifestyle, including physical activity as well as on quality of life.

The participants having an appointment for a routine health check by a PCP who is assigned to the intervention group will receive the risk prediction model “The German Diabetes Risk Score” (GDRS) in addition to the baseline questionnaire.

The GDRS is based on a prospective cohort study (European Prospective Investigation into Cancer and Nutrition [EPIC]-Potsdam study), including 25,000 people and has been externally validated several times [4, 12–14]. It was shown that the GDRS provides good prediction of type 2 diabetes incidence according to the receiver operating characteristic curve (ROC-AUC): [95%-KI]: 0.86 [0.84–0.87]) [13], even in other German cohorts (ROC-AUC between 0.70–0.87) [12, 14]. The GDRS focuses on modifiable non-invasive risk factors and consists of eleven questions on age, height, waist circumference, hypertension, physical activity, smoking status, intake of whole-grain bread, intake of red meat, coffee consumption, and two questions regarding the family history of diabetes (parents and siblings) to predict the five-year diabetes risk. In addition, the GDRS contains a visual presentation of the individual diabetes risk and short recommendations to enhance healthy lifestyle. The filled diabetes risk score will be used in the counseling interview with the PCP at the end of the health check as a practical guide to discuss individual tailored preventive strategies.

### Control

All participants having an appointment for a routine health check by a PCP who is allocated to the control group will receive solely the routine health check and fill out the regular baseline questionnaire.

### Further study course

After the health check, participants will be followed for 12 months. They receive questionnaires after six and 12 months which can be filled in online by using a personalized link or otherwise paper based. The questionnaires include, among others, questions about their lifestyle, current status of health and the counseling session at the end of the routine health check. Differences between the questionnaires in the two groups are related to the use of a diabetes risk score in the intervention group.

Anthropometric measures of participants are objectively measured by PCPs at the first day of the health check at baseline and via self-report at follow-up assessments. As the waist circumference is overestimated by patients without initial guidance by the PCP (in average about 6 cm) [15], patients will be trained at the baseline appointment in order to ensure accurate measurement. After the baseline medical appointment, participants take the measuring tape for the follow-up questionnaires at six and 12 months. Laboratory tests (blood and urine) will be performed at baseline and updated in case of another doctor’s visit in the follow-up within a maximum time frame of 3 years.

In addition to the measurements taken from the participants, measurements will also be taken from the PCPs, e.g., on PCP’s workflow in the medical office, implementation of lifestyle prevention strategies, and acceptance of the application of a diabetes risk score in the primary health care setting. PCPs will receive two general questionnaires at baseline and after inclusion of the target number of participants. Furthermore, one questionnaire is filled in by the PCP after each health check with a study participant. This questionnaire includes questions about the content of consultation at the end of the routine health check, shared decision making, as well as obtained clinical data. At least 1 year after inclusion of all participants, clinical data of each participant will be updated by the PCPs.

**Fig. 1:**
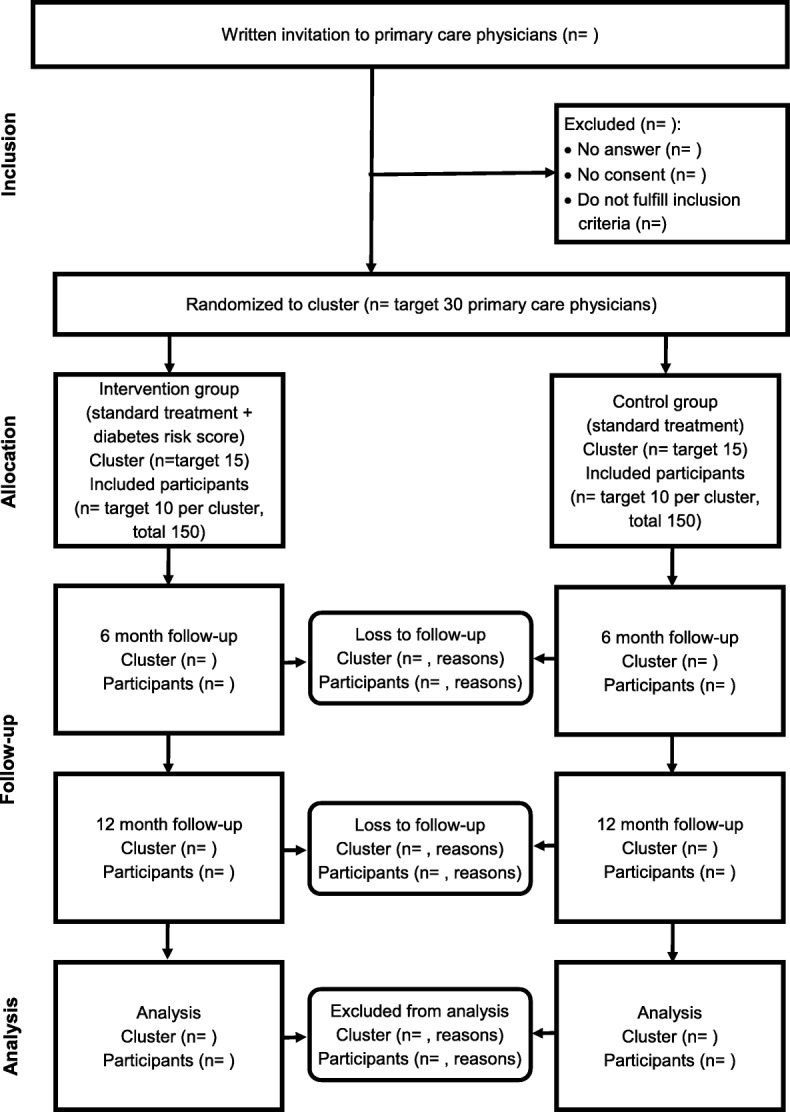
Flow diagram of the study

Figure 1 shows the flow diagram of the study. Table 1 gives an overview on participant’s self-reported measures, Table 2 lists measures assessed by PCPs. The questionnaires, which are compilations of validated questionnaires, and adopted questions from previous studies or systematic reviews, were developed and tested by an interdisciplinary team, and were validated using cognitive interviewing techniques with a sample of six PCPs and six persons. Furthermore, questionnaires and data collection process were tested in a pilot study was conducted including four physicians and 33 participants.

**Table 1:** Self-reported measures assessed by participants

Measures		Brief Description	Time of assessment		
			Baseline (Check-up 35 visit)	Follow-up 6 months	Follow-up 12 months
Sociodemographic and anthropometric characteristics^c^		Age, sex, height, first language, family status, level of education, employment status	✓ IG, CG		
		Weight, smoking status, presence of hypertension	✓ IG, CG	✓ IG, CG	✓ IG, CG
Neighborhood environmental perception and dog ownership^c^		Physical activity is correlated with several environmental factors, thus we use the first seven questions of the validated European questionnaire “ALPHA” [30] to assess environmental perceptions. For the same reason, we included a question about dog ownership derived from another study by Panter et al. [31].	✓ IG, CG		
Social residential environment^c^		Physical activity is correlated with assistance from family and friends, thus, four questions are included, rated on a 4-point Likert scale (completely agree - completely disagree) adopted from another study on physical activity and residential environment [32].	✓ IG, CG		
Physical activity^a^		International Physical Activity Questionnaire (IPAQ)-Short Form [16], intentionally mostly used validated questionnaire to assess physical activity of the last seven days. The questionnaire consists of seven questions and has acceptable measurement properties [16].	✓ IG, CG	✓ IG, CG	✓ IG, CG
Counseling process^b^	counseling content	Questions about the content of consultation regarding the lifestyle factors weight reduction, change of diet, physical activity and smoking cessation. Discussion of lifestyle factors, target agreements, received information, referral to experts/counseling centers. Questions were obtained from a study on diabetes screening by Hussain et al. [33]		✓ IG, CG	
	future medical appointments	Question, if a medical appointment was arranged and observed. This aspect was identified in an earlier focus group with PCPs in the planning phase of the trial.			✓ IG, CG
Proportion of shared decision making^b^		Modified questionnaire to assess shared decision making for diabetes prevention opportunities including six questions. Five questions are ranked on a 6-point Likert scale (completely disagree-completely agree), and one overall question. The original questionnaire PEF-FB-9 [34] was developed and validated for clinical encounters in the primary health care setting and people with an existing medical decision.	✓ IG, CG		
Motivation and/ or change of lifestyle^b^		For assessment of readiness to change lifestyle (weight reduction, regular physical activity, healthy diet, smoking cessation) we use the stage of change model developed by Prochaska et al. [35–38], allowing allocation of individuals to different stages of readiness for change (from precontemplation through contemplation, preparation, and action to maintenance)	✓ IG, CG	✓ IG, CG	✓ IG, CG
Changes of medical condition^b^	General medical condition	Self-rated overall health question with five answer alternatives “very good”, “good”, “fair”, “bad”, “very bad” suggested by World Health Organization and already used in a national representative German study (DEGS1) [39, 40]	✓ IG, CG	✓ IG, CG	✓ IG, CG
	Depression and anxiety	The Hospital Anxiety and Depression Scale (HADS) is a validated questionnaire to assess anxiety and depression. The questionnaire consists of 14 items, seven anxiety and seven depression items and showed good reliability and validity in several settings including general population [41, 42].	✓ IG, CG	✓ IG, CG	✓ IG, CG
Changes in diabetes risk^b^		Changes of diabetes risk in the intervention group will be analyzed, as well as differences between the groups at follow-up. The German Diabetes Risk Score [13] is a validated non-invasive risk score to predict the five year risk of developing diabetes. It consists of eleven questions with modifiable (dietary habits, physical activity) and non-modifiable factors (e.g. age, height, family history of diabetes). Only the intervention group receives the original questionnaire at baseline. In the follow-up the questions of the risk score are included separately without any risk output in both groups.	✓ IG	✓ IG, CG	✓ IG, CG
Perceived diabetes risk^b^	Memory of diabetes risk	Participants are asked, if they can remember in which risk category they were assigned by the German Diabetes Risk Score at baseline. This question reflects the perceived importance of diabetes risk and intensity of awareness. The question is adopted by a study from Godino et al. [9].		✓ IG	✓ IG
	Perceived diabetes risk	Questions about the perceived risk of already having type 2 diabetes and perceived risk of developing type 2 diabetes in the next five years are included. The questions have been already used in other studies [9, 43, 44].		✓ IG, CG	✓ IG, CG
Acceptance of diabetes risk score^b^		Different questions regarding the understandability, length, and acceptance of the German Diabetes Risk Score. Questions were adapted from other studies, aspects synthesized by Dhippayom et al. [5]		✓ IG	

*CG* control group, *IG* intervention group, *PCPs* primary care physicians, ^a^primary outcome, ^b^secondary outcome/other outcomes, ^c^general measures/confounder measures

**Table 2:** Measures assessed by PCPs

Measures		Brief Description	Time of assessment			
			Baseline at inclusion	For each participant	Follow-up after recruiting completed	One year after inclusion of all participants
Demographic statistics and information about PCP and medical office^c^		Sex, age, date of establishment, medical degree, additional degrees, type of medical office (community practice, solo practice etc.)	✓ IG, CG			
		Number of patient contacts and health checks (Check-up 35) per quarter	✓ IG, CG		✓ IG, CG	
Counseling process^b^	Motivation and implementation of prevention strategies in general	We use a set of questions from the validated nationwide survey on modifying health behavior to prevent cardiovascular diseases among German PCPs (ÄSP-Study) [45]: a) Frequency of provision of lifestyle advice of weight reduction, increasing physical activity, achieving a healthy diet, quitting smoking, and some further measures of risk factors on a 5-point Likert scale (no provision - provision to all patients) b) perceived importance of reduction of risk factors is rated on a 4-point Likert scale (very important - not important at all) c) PCPs self-rated competence to motivate patients changing lifestyle is also rated on a 4-point Likert scale (very high - very low) d) The level of PCPs’ agreement to statements regarding attitudes, self-competence, and self-efficacy of PCPs is rated on a 4-point Likert scale (completely agree - completely disagree) e) Cooperation’s to other institutions and experts regarding prevention In addition, questions on obstacles of counseling were derived from two qualitative studies on lifestyle counseling in diabetes care and obese patients [46, 47]	✓ IG, CG		✓ IG, CG (solely a and c)	
	Content of counseling with participant	After each routine health check with a study participant PCPs are asked about the content of counseling (counseling, information material, and referral to expert) regarding weight reduction, increasing physical activity, achieving a healthy diet and quitting smoking using tick boxes. The framework is adopted from two studies [45, 48].		✓ IG, CG		
	Future medical appointments	Question, if a medical appointment was arranged and when. This aspect was identified in an earlier focus group with PCPs in the planning phase of the trial.		✓ IG, CG		
Proportion of shared decision making^b^		Modified questionnaire to assess shared decision making for diabetes prevention opportunities including six questions. Five questions are ranked on a 6-point Likert scale (completely disagree-completely agree), and one overall question. The original questionnaire PEF-FB-doc [49] is the pendant to PEF-FB-9 and was developed and validated for clinical encounters in the primary health care setting and people with an existing medical decision.		✓ IG, CG		
Acceptance of diabetes risk score^b^	Use of diabetes risk scores	Question adopted from the ÄSP-Study [45] about which diabetes risk scores are known and used in everyday practice.	✓ IG, CG			
	Attitude towards diabetes risk scores	Different questions regarding the usefulness, validity, understandability and acceptance towards the diabetes risk score. Questions were adapted from a qualitative study by Müller-Riemenschneider et al. [10] and a systematic review by Dhippayom et al. [5]. Usefulness is rated on a 5-point Likert scale (not useful – very useful), all other questions are rated on a 4-point Likert scale (completely agree - completely disagree)	✓ IG (CG: question about usefulness only if PCP knows diabetes risk scores)		✓ IG, CG	
Anthropometric characteristics of participants^b^		The PCP measures waist circumference, weight, and height from each participant objectively. In the IG, measures are used to generate diabetes risk score.		✓ IG, CG		
Clinical data from routine health check (Check-up 35)^c^		Routine health check results (medical examination, lab tests, diagnoses) are collected, the questions are derived from a study that was conducted in the same health setting (Esther-Study) [48]. At the last survey, prescription of diabetes medication is asked in addition.		✓ IG, CG		✓ IG, CG

*CG* control group, *IG* intervention group, *PCPs* primary care physicians, ^a^primary outcome, ^b^secondary outcome, ^c^general measures/confounder measures

To ensure adherence to the protocol, the study personnel will arrange a face-to-face meeting with each PCP at the beginning of the trial. At the meeting, all aspects of the study are discussed, including the recruitment of participants, handling of the questionnaires and conduct of the health check with or without the application of the risk prediction model. In addition, a video explaining all aspects of the study will ensure adherence to the protocol during the time of the study.

### Primary outcome measure

- Difference in physical activity at 12 months after the routine health check between the groups.

We will assess physical activity with the internationally validated questionnaire International Physical Activity Questionnaire Short Last 7 Days Format (IPAQ-SF), which has been shown to be a reliable and valid tool to obtain comparable estimates of physical activity [16]. The questionnaire includes questions on different levels of physical activity (walking, moderate- and vigorous-intensity activities), their frequency (days per week in the last 7 days) and duration (minutes per day, at least 10 minutes of activity). In our study, physical activity will be measured at baseline, and at six and 12 months follow-up. Low, moderate, and vigorous physical activity will be calculated as the sum of the metabolic equivalent of task (MET) minutes per week, according to the standard official manual [17], in which all minutes of activity during the last 7 days are multiplied with the respective metabolic rate which is defined as 3.3 for walking, 4.0 for moderate physical activity and 8.0 for vigorous physical activity. This primary outcome measure was selected as it is a patient relevant outcome that reflects possible lifestyle changes, starting with the application of the diabetes risk score in the counselling interview of the routine health check. This can improve awareness of participants regarding risk factors associated with diabetes, which may lead to a healthier lifestyle.

### Secondary outcome measures

- Improvement in the counseling process regarding preventive strategies on balanced nutrition, body weight reduction and smoking secession,
- improvement of shared decision making,
- change in BMI
- improved motivation to change lifestyle,
- change in quality of life, and change in level of depression and anxiety,
- change of the perceived risk of developing diabetes,
- acceptance of the application of a diabetes risk score for routine use in clinical practice.

Other Pre-specified Outcome Measures

- Change in individual diabetes risk at 6 and 12 months in the intervention group and difference of individual diabetes risk between 6 and 12 months follow-up between the groups.

All secondary and other outcome measures will be obtained via self-report questionnaires. Detailed information of the measures and time points of assessment can be found in Tables 1 and 2.

### Sample size

The primary endpoint will be the change in physical activity after 12 months follow-up between the groups, measured by the IPAQ short form questionnaire as the sum of MET minutes per week [16, 17]. The study by Yates et al. [18] had a comparable study design, hence, we use this study as the reference for sample size estimation. In the Yates study, participants had an interquartile range of 1180–4719 MET minutes per week, which corresponds approximately to a standard deviation of 2665 MET minutes per week [19]. Our pilot study conducted prior to the main study showed similar results on the standard deviation of physical activity after 3 months. The study by Yates et al. yielded an observed group difference of 2836 MET minutes per week, however, with a more intensive program. Thus we set our minimally clinically relevant effect to 1050 MET minutes per week including all types of activities, which corresponds to a group difference of moderate activity five times a week for 30 min and walking for three times a week for 45 min. As intracluster correlation coefficients (ICCs) in cluster randomized trials in primary care were observed to be small, we assume an ICC of 0.01 [20]. Together with a projected cluster size of ten participants per cluster, this leads to a design effect of 1.09 [21]. Finally, to detect a difference of 1050 MET minutes per week with a standard deviance of 2665 MET, a design effect of 1.09, an expected dropout rate of 30%, a power of 80%, and a significance level of 5% of a cluster-adjusted t-test, 300 participants, split up on 15 PCPs per study group are needed.

### Recruitment strategy

To ensure adequate participant enrollment, the study team will maintain close contact to the PCPs. This includes communication per email or telephone and the collection of the baseline questionnaires at the PCP’s practices regularly. Hereby, the study personnel can solve possible failures in the study process and resolve missing values in the questionnaires promptly on-site.

### Randomization, allocation concealment and blinding

To avoid dilution of the intervention effect, e.g. by participants exchanging information in the waiting room, the study was designed as a cluster trial, in which the PCPs are the unit of randomization. PCPs who are willing to participate are randomly allocated to intervention or control group by a blinded clinical data manager without knowledge of the PCPs. Minimization technique including biased coin design (*p* = 0,7) (Software: MinimPy 0.3 [22]) will be used to balance among the following properties of PCPs: residency (general medical vs. internal medicine), additional training in diabetology (yes or no) and the socio-economic environment of the practices, based on statistical data about unemployment rates in the defined region. It is not possible to blind PCPs according to study group, because of PCP networks and knowledge exchange. In addition, it is not possible to blind the researchers involved in the project management and implementation of the study, but participants and researchers involved in the analysis will be unaware of the assigned study group.

### Data management, quality assurance and data protection

All participants’ questionnaires filled in at baseline and all PCP questionnaires are paper-based and will be collected in a study folder at the medical practice. A continuous monitoring is performed by the study personnel, in which the questionnaires are checked for completeness and plausibility at the medical practice every quarter. Follow-up questionnaires for participants are sent and completed via the online tool REDCap [23], a secure web application for building and managing online surveys and databases, and otherwise by postal service. In case of missing or implausible values in the questionnaires, PCPs and participants are contacted up to three times for completion or correction. Each PCP and participant receives a unique numeric identifier when participating in the study. Personal information will be stored separately in locked filing cabinets, and the link between personal information and obtained data is only accessible by the study coordinator. All paper-based data will be doubly entered by trained personnel to assure high data quality. Personal contact to PCPs and participants shall prevent premature drop-out. Furthermore, monetary incentives are given to PCPs and participants. All data obtained in the study are subject to strict data protection regulations.

### Data analysis and dissemination of results

To prevent systematic bias, analysis will be conducted using the intention-to-treat principle. Missing values will be handled by multiple imputation. For the purpose of possible bias due to the imputation of missing values, the analyses will also be conducted as complete case analysis and differences of both approaches will be discussed (sensitivity analysis) [24]. For the analysis of the intervention effect (primary outcome at 12 months, secondary outcomes at different time points) hierarchical or mixed models will be applied for primary and secondary endpoints, including a random intercept to adjust for the cluster effect, the respective baseline value, covariates used for minimization and individual covariates age, sex and smoking status of participants at baseline. We will use generalized linear mixed models with the identity link for continuous and the logit link for binary outcomes.

In terms of the primary endpoint, we define a difference of Δ = ± 300 MET / min per week or smaller as a clinically irrelevant difference. That is, if we observe the 95% confidence interval for the primary endpoint lying completely within [− 300; 300], we judge the intervention to be ineffective. The study will be reported according to the CONSORT statement for the reporting of cluster randomized control trials [25].

### Ethics

This study gained full ethical approval from the ethics committee of the Heinrich Heine University Düsseldorf in June 2017 (Reference-No: 5540).

## Discussion

This pragmatic cluster randomized controlled trial will enhance our knowledge on the impact of diabetes risk scores under real-life conditions in the primary health care setting. Especially non-invasive risk prediction models without laboratory measurements are free of charge and easy to apply, as the participants can answer the questions themselves to obtain their personal risk of diabetes within the next years, thus, these questionnaires have the potential to increase efficiency of identifying persons at risk. So far, a number of risk prediction models are ready to use and the utilization is required in current guidelines [3, 5, 7]. Unfortunately, PCPs use diabetes risk prediction models only marginally because of various barriers, e.g. lack of time and reimbursement, as well as patient’s cultural or language issues [5, 10].

A currently published randomized clinical trial by Godino et al. [8, 9] on effects of communicating the risk of type 2 diabetes was the first controlled study examining effects of the application of a genetic or phenotypic risk score compared to standard lifestyle advice on the health behaviors of participants recruited from the Fenland Study (UK). The study showed no significant intervention effects on physical activity and self-reported diet, weight, worry and anxiety. Solely the perceived risk was more accurate in those participants that received information about their risk estimate. Some factors need to be taken into account when interpreting these results: study participants were recruited from the Fenland study, which is a cohort of relatively young and slightly overweight individuals before the onset of chronic diseases [8, 26]. Furthermore, it is unclear if possible dilution effects appeared in the counselling effects, because Godino et al. did not declare if members of the study team delivered the interventions separately. Finally, participants were not blinded to the intervention they received, meaning that participants in the control group might have used phenotypic risk scores available on the internet to obtain their personal risk estimate.

However, there are also some limitations in our study design. We use only self-reported measurements to assess lifestyle changes, e.g. physical activity. It is well known that the IPAQ somewhat overestimates physical activity [27], but other budget-friendly objective measurements, for example pedometer, measures only the distance travelled by feet and often fail to record slower, shuffling steps, which is often the case in older patients [28]. Moreover, objective measurements with pedometers or accelerometers may motivate patients to increase physical activity [29], an aspect, which could also lead to a dilution of the intervention effect in our study. The IPAQ is cost-effective, can assess discrete categories of physical activity, and is the most used questionnaire to measure physical activity worldwide. Therefore, the results are comparable to other studies in this field. Because we are interested in intraindividual change of physical activity 6 and 12 months after the routine health check, we believe the use of a self-reported instrument in our study is acceptable. Another potential limitation is that the questions of the GDRS are also filled in by patients themselves. This could lead to an underestimation of diabetes risk [4]. However, all questions of the GDRS are short and easy answerable. All participants will be trained on measurement of the waist circumference at the day of routine health check and receive corresponding instructions for all measurements they need to fill in the questionnaire. In addition, a number of validation studies showed that the GDRS predicted insulin resistance with acceptable accuracy (ROC-AUC between 0.70–0.87) [4, 12, 14], thus, we are convinced that the individual diabetes risk can be measured adequately by the GDRS. Although participants are blinded concerning the intervention, in cannot be excluded that people in the control group may use a diabetes risk score which are accessible for public on the internet. However, this would only lead to an underestimation of the intervention effect.

Our study, which is designed as a blinded pragmatic cluster randomized trial, will be conducted in close cooperation with PCPs to assess possible intervention effects of the application of a risk prediction model in a real world setting without possible dilution effects in the counselling process. Moreover, PCPs are the first point of contact in which the relationship between the PCP and the participant is almost always characterized by trust. The study participants will have a higher risk to develop diabetes, based on the inclusion criterion BMI ≥27 kg/m^2^. Besides that, we will pursue a pragmatic approach that will lead to more heterogenic groups, especially regarding age and medical condition. Another important aspect is that we are not only interested in measurements from the participants, but also from the PCPs. This will enable us to identify the habits, possible changes in the counseling process and the acceptance of the application of diabetes risk prediction models. In summary, our study is slightly different to the study by Godino et al. [8] with a more pragmatic approach and additional assessments of outcomes assessed by PCPs that may have an impact on the outcomes in this trial.

The application of diabetes risk prediction models may lead to more targeted counseling on preventive opportunities, increase risk-awareness, and activates people’s motivation to change lifestyle. From the public health perspective, the study results can contribute to future development of diabetes prevention strategies, meaning that in case of positive effects, diabetes risk prediction models could be regularly integrated in PCPs every day practice. Overall, this easy applicable, low cost intervention may lead to cost reductions for the health care system.

## Abbreviations

BMI: Body Mass Index
GDRS: German Diabetes Risk Score
HbA1c: Glycated hemoglobin A1c
ICC: Intracluster correlation coefficient
IPAQ-SF: International Physical Activity Questionnaire Short Last 7 Days Format
MET: Metabolic equivalent of task
oGTT: Oral glucose tolerance test
PCPs: Primary care physicians
ROC-AUC: Receiver operating characteristic area under the curve
